# Isolation, Passage, and Pathogenicity of a Newly Isolated *Lawsonia intracellularis* Strain From Hubei, China

**DOI:** 10.1155/tbed/2501719

**Published:** 2025-07-01

**Authors:** Rui Xie, Yajuan Luo, Changjiang Peng, Wenqing Wu, Yucong Cui, Xinru Sun, Lin Hua, Bin Wu, Zhong Peng

**Affiliations:** ^1^National Key Laboratory of Agricultural Microbiology, College of Veterinary Medicine, Huazhong Agricultural University, Wuhan, China; ^2^Hubei Hongshan Laboratory, Wuhan, China; ^3^The Cooperative Innovation Center for Sustainable Pig Production, Frontiers Science Center for Animal Breeding and Sustainable Production, Wuhan, China; ^4^Twins' Group Co. Ltd., Nanchang, China

**Keywords:** isolation, *Lawsonia intracellularis*, passage, pathogenicity, pigs

## Abstract

*Lawsonia intracellularis* is the causative agent of porcine proliferative enteropathy (PPE)—a disease of great economic impact to the global pig industry, but the isolation and continuous passage culture of this bacterial species is very difficult which limits the development of inactivated or live vaccines. While China is the largest pig rearing country in the world, only one study has reported the isolation of *L*. *intracellularis*. In this study, we examined 1574 ileal samples collected from 10 slaughterhouses in Hubei Province, China, and obtained 104 samples tested positive for *L. intracellularis*. From these positive samples, we successfully isolated a *L. intracellularis* strain designated LI-HuB23, which could be continuously passaged in IEC-18 cells. The successful isolation of LI-HuB23 was confirmed by transmission electron microscopy (TEM), indirect immunofluorescence assay (IFA), PCR amplifying, and Sanger sequencing of the marker gene (*aspA*). LI-HuB23 exhibited stable proliferation over 10 passages and it was still being passaged for over 30 generations. Oral inoculation of 28-day-old pigs with LI-HuB23 containing 6.9 × 10^8^ bacterial microorganisms induced loose stools and watery diarrhea between Days 14 and 28 postinfection. Challenging pigs showed an average daily gain (ADG) lowered than the control pigs (206.05 ± 23.48 g/day vs. 241.43 ± 16.78 g/day). All challenging were serologically positive for *L. intracellularis* IgG at 21 days postinoculation. Histological examination detected crypt hyperplasia, characterized by a reduction in goblet cells within the hyperplastic crypts. Colonization of *L. intracellularis* in ileal crypts was confirmed by immunohistochemical examination.

## 1. Introduction

Porcine proliferative enteropathy (PPE), also known as porcine ileitis, is a significant enteric disease caused by the intracellular bacterium *Lawsonia intracellularis* [1–3]. The clinical manifestations of PPE are generally classified into acute, chronic, and subclinical forms, each with distinct pathological features [4]. The acute form, often referred to as hemorrhagic proliferative enteropathy, mainly affects pigs aged 4–12 months and is characterized by severe clinical signs, including bloody diarrhea, tarry red stools, melena, and in some cases, sudden death [5]. The chronic form, commonly identified as porcine adenomatous disease, affects pigs between 6–20 weeks of age, presenting with intermittent diarrhea, anorexia, and stunted growth, which ultimately leads to impaired growth uniformity within affected cohorts. The subclinical form, which represents the most common manifestation of *L. intracellularis* infection, is often asymptomatic or characterized by subtle signs, making it challenging to diagnose clinically [4, 6]. Despite the absence of obvious symptoms, subclinical infections result in reduced growth performance and significant economic losses, primarily due to poor growth uniformity within the herd [7, 8].

*Lawsonia intracellularis* is an obligate intracellular, gram-negative bacterium, with dimensions ranging from 1.25 to 1.75 μm in length and 0.25 to 0.43 μm in width. This bacterium is nonspore-forming, microaerophilic, and typically exhibits a bacillary morphology, which can be either curved or straight [1]. Despite extensive research, *L. intracellularis* cannot be cultured on conventional bacteriological media and necessitates a microaerophilic environment for successful in vitro cultivation [3]. The culture and growth of *L. intracellularis* depend on the use of specific eukaryotic cell lines, including IEC-18, Int 407, PK-15, IPEC-J2, and McCoy cells [3, 9, 10]. Moreover, the complex and highly contaminated intestinal milieu in which *L. intracellularis* resides also makes the isolation and maintenance of this bacterial species under laboratory conditions a considerable challenge, limiting further research and the development of control strategies against *L. intracellularis* at the global level [11].

China is the largest pig-rearing and pork-consuming country globally and the pig industry plays a pivotal role in the nation's economic infrastructure. Epidemiological investigations have shown that *L. intracellularis* infection is common in pigs in China, with a farm positivity rate as high as 93.6% [12]. However, only one study has reported the isolation of *L. intracellularis* from pigs in China [13], marking a milestone in isolating the Chinese epidemic clinical strains, but additional bacterial recoveries are necessary. In this study, we isolated a strain of *L. intracellularis*, designated LI-HuB23, from ileitis-positive samples collected from slaughtered pigs in Hubei Province using the IEC-18 cells (ATCC CRL-1589). LI-HuB23 demonstrated stable propagation and passage in the cells for over 30 generations. Oral inoculation of 28-day-old pigs with cell-cultured LI-HuB23 successfully induced clinical manifestations of PPE. These results highlight the potential of LI-HuB23 as a promising candidate for the development of vaccines against PPE.

## 2. Materials and Methods

### 2.1. Sample Collection and qPCR Detection

From 2023–2024, a total of 1574 pig ileal samples were collected from 11 slaughterhouses in Hubei Province, China. Following collection, intestinal mucosa samples (50–100 mg) were scraped and homogenized in 1 mL of sterile saline, followed by centrifugation at 200 × *g* for 2 min to collect the supernatants. DNA extraction was performed using a commercial nucleic acid extraction kit (Vazyme Biotech, Nanjing, China) and utilized as templates to examine the nucleic acids of *L. intracellularis* using qPCR assays. The primers were designed to target the *aspA* gene (forward: CCTTGGAGGTAAATTGATTTCTCC and reverse: ATGTTCAGCTTTCTGGTGTTCTTA) and probe (FAM-TCCACAGCGAGGACCACTTGAGA-TAMRA), which have been widely used for the identification of *L. intracellularis* [14–16]. Additionally, the full length of the *aspA* gene of *L. intracellularis* strain N343 (GenBank accession no. CP004029) was synthesized and cloned onto the pUC-57 vector to generate the standard plasmid. DNA copy numbers were calculated using the formula: *Ct* = −3.421 × 1 g (DNA copy number) + 42.89 (*R*^2^ = 0.9986 and efficiency = 96.02%).

### 2.2. Cell Line and Culture Conditions

Rat intestinal epithelial cells (IEC-18; ATCC CRL-1589) were cultured in Dulbecco's modified eagle medium (DMEM; Gibco, USA) supplemented with 10% fetal bovine serum (FBS; Gibco, USA) and without antibiotics. The cells were passaged every 2 days at a ratio of 1:4 using 0.25% trypsin (Gibco, Thermo, Waltham, USA). For infection, cells were seeded at a density of 3 × 10^4^ cells/mL in DMEM containing 5% FBS and no antibiotics 1 day prior to inoculation. Uninfected control cells were maintained at 37°C in a humidified atmosphere with 5% CO_2_.

### 2.3. Isolation of *Lawsonia Intracellularis*

*Lawsonia intracellularis* strains were isolated from positive samples using IEC-18 cells, following previously published methods [13, 17, 18]. Briefly, the mucosal specimens were homogenized in phosphate-buffered saline (PBS, pH 7.4) containing 1% trypsin (Gibco, Thermo, Waltham, USA) using a QIAGEN TissueLyser II (QIAGEN, Hilden, Germany), followed by incubation at 37°C for 35 min. The homogenates were mixed with an equal volume of sucrose–potassium glutamate (SPG) solution (0.218 M sucrose, 0.0038 M KH_2_PO_4_, 0.0072 M K_2_HPO_4_, and 0.0049 M potassium glutamate; pH 7.0) supplemented with 10% FBS (Gibco, USA). Subsequently, the mixture was filtered using membranes with decreasing pore sizes (100-, 70-, 40-, 1.2-, 0.8-, and 0.65-μm). The filtrates were diluted in DMEM (Gibco, USA) containing 7% FBS at a volume ratio of 1:10. The dilutions were inoculated into IEC-18 cells at 30% confluence and maintained at 37°C in a tri-gas chamber (8% O_2_, 8.8% CO_2_, and 83.2% N_2_). Three hours postinfection, the medium was replaced with fresh DMEM supplemented with 5% FBS, neomycin, vancomycin, and amphotericin B and refreshed every 2–3 days. The infection medium was refreshed every 2–3 days. Bacterial cultures were harvested at 7 days post medium replacement and subsequent passaging were conducted as previously described by Lawson et al. [3].

### 2.4. Phylogenetic Analysis

The full-length nucleotide sequence of *aspA* (1416 bp) was amplified using PCR with a pair of designed primers (forward: 5′-TTACATGACTTTCATTTTGA-3′ and reverse: 5′-ATGCATGAATTCCGTAAAGA-3′) in a 25 µL reaction mixture containing 12.5 µL of 2 × Taq Master Mix (Vezamy, Nanjing, China), 10 µM of each primer, 2 µL of *L. intracellularis* genomic DNA, and 8.5 µL of nuclease-free water. Cycling conditions were initial denaturation at 95°C for 5 min, followed by 35 cycles of 95°C for 15 s, 45°C for 15 s, 72°C for 1 min, and a final extension at 72°C for 10 min. Sanger sequencing (Beijing Tsingke Biotech, China) was conducted on the PCR products to obtain the amplified sequence, which was compared to the nucleotide sequences of *L. intracellularis* aspA publicly available in NCBI using Clustal W. A neighbor-joining tree was generated based on the sequence comparison results using MEGA 11, with 1000 bootstrap replicates [19].

### 2.5. Preparation of a Monoclonal Antibody Against *Lawsonia Intracellularis*

Monoclonal antibodies were prepared following the methods reported by Guedes and Gebhart [20] and Boesen et al. [21]. Briefly, inactivated *L. intracellularis* (1 × 10^8^ bacterial microorganims) was mixed with QuickAntibody adjuvant (Biodragon, Beijing, China) at a volume ratio of 1:1, which was injected intramuscularly into 8-week-old specific pathogen-free (SPF) Balb/c mice (purchased from the Laboratory Animal Center at Huazhong Agricultural University). At 21- and 35-days postinjection, a booster immunization through the same routine was given, respectively. At 38-days postinjection, the mice were euthanized and spleens were collected. Spleen cells were fused with SP2/0 myeloma cells (ATCC CRL-1581) using 50% polyethylene glycol hybridomas (Thermo, Waltham, USA). Fused cells were screened in hypoxanthine–aminopterin–thymidine (HAT) medium (Thermo, Waltham, USA). HAT-resistant clones were further screened using IFA to identify the cells which can secret *L. intracellularis*-specific antibodies. The cells were purified using the limiting dilution (1 cell/well) method. Mouse experiment was approved by the Animal Experiment Ethics Committee of Huazhong Agricultural University.

To prepared monoclonal antibodies were used to examine the *L. intracellularis* strain isolated in this study, the vaccine strain B3903 (Boehringer Ingelheim Enterisol Ileitis), IEC-18 cells (ATCC CRL-1589), McCoy cells (ATCC CRL-1696), *Salmonella choleraesuis* (C78-1, China institute of veterinary drug control, Beijing, China), and/or *Escherichia coli* O157:H7 (EDL933) using western blot analysis. In brief, bacterial strains or cells were lysed through sonication and were lysed in sodium dodecyl sulfate (SDS). Total proteins were separated using SDS-polyacrylamide gel electrophoresis (SDS-PAGE) and were transferred onto polyvinylidene difluoride (PVDF) membranes. The membranes were then blocked at 4°C overnight in PBS containing 1% skimmed milk. After washing using PBST, the membranes were incubated with the prepared monoclonal antibody (1:5000 dilution) for 2 h at room temperature. Membranes were then incubated with horseradish peroxidase (HRP)-conjugated goat anti-mouse IgG (1:1000 dilution; Proteintech, Wuhan, China) and were finally stained using diaminobenzidine and hydrogen peroxide solution (Beyotime, Shanghai, China) according to the manufactory instructions.

### 2.6. Indirect Immunofluorescence Assay (IFA)

The indirect IFA was also conducted to examine *L. intracellularis* strains using the prepared monoclonal antibody. In brief, the *L. intracellularis* strain isolated in this study and/or the strain B3903 (1 × 10^7^ bacterial microorganisms) was inoculated into IEC-18 monolayers and were incubated at 37°C for 5 days in a tri-gas chamber. The cells were fixed with pre-cooled methanol for 15 min and washed three times using PBS, followed by incubating with the prepared monoclonal antibody (C17) at 37°C for 1 h. Bacteria incubated with a commercially purchased monoclonal antibody against *L. intracellularis* (BIO 323; Bio-X Diagnostics, UK) at the same conditions were used as controls. After incubation, the cells were washed using PBS and were incubated with a Cy3-conjugated goat anti-mouse IgG (1:500 dilution; Proteintech, Wuhan, China) in dark for 1 h at 37°C. Finally, the cells were stained using 4,6-diamidino-2-phenylindole (DAPI; Beyotime, Shanghai, China) for 10 min at room temperature in the dark and were examined using an inverted fluorescence microscope (Nikon, Japan).

### 2.7. Transmission Electron Microscopy (TEM)

To prepare bacterial samples for TEM examination, the supernatants of cell culture were centrifuged at 12,000 rpm, 4°C, for 10 min. The pellets were washed three times using PBS and were fixed using 2.5% glutaraldehyde at 4°C for 4 h. The samples were then adsorbed onto copper grids and stained using 1% sodium phosphotungstate for 30 s. The grids were subsequently examined using a transmission electron microscope (Hitachi, Japan).

### 2.8. Bacterial Titering

To determine bacterial titer, *L. intracellularis* was inoculated into IEC-18 monolayers in different wells of a 96-well plate (Corning, Corning, USA). The plate was incubated at 37°C for 6 days in a tri-gas chamber. A medium replacement was given at the third days postinoculation. *Lawsonia intracellularis* was detected using IFA and infected cells were enumerated. The TCID_50_ values were calculated using the Reed–Muench method [22], with eight replicates per dilution.

### 2.9. Animal Experiments

To assess the virulence of the *L. intracellularis* isolated in this study, ten 28-day-old pigs (purchased from the Laboratory Animal Center at Huazhong Agricultural University) were randomly divided into two groups and each group contained five pigs. Before experiment, blood and fecal samples were collected to confirm *L. intracellularis*-negativity using IFA and qPCR, respectively. Afterwards, each pig in one group (LI-challenging group) was orally inoculated with *L. intracellularis* (6.9 × 10^8^ bacterial microorganisms in 10 mL SPG buffer), while pigs in the other group received oral administration of 10 mL SPG buffer per pig (SPG-treated group). Body weights were recorded once at 0-, 7-, 14-, 21-, and 28-days postinoculation. Fecal samples were collected from each pig twice every week postinoculation to examine the bacterial DNAs using qPCR. Blood was collected from each pig every 7-days postinoculation to detect the serum antibody using the IFA method described below. At 18-days postinoculation, one pig from LI-challenging group was euthanized and dissected. The ileum was collected for histological and immunohistochemical detection. All remaining pigs were euthanized and dissected at 31-days postinoculation. Fecal scores were formulated reference to Joens et al. [23]: Grade 0 = normal feces, Grade 1 = soft feces, Grade 2 = watery diarrhea, Grade 3 = hemorrhagic diarrhea, and Grade 4 = mortality.

Antibodies in pig serum induced by *L. intracellularis* were determined using the IFA method described by Guedes et al.[24]. *In* brief, *L. intracellularis* infected IEC-18 cells in wells of a 96-well plate were fixed using cold methanol at the 6 days post bacteria-infection. After washing three times using PBS, 100 µL of distilled water was added into each well and the plate was incubated at 37°C for 10 min. Serum samples were diluted in PBS containing 5% BSA at a volume ratio of 1:15, and a series of twofold-dilutions were added into the wells, followed by incubation at 37°C for 30 min. After washing three times using PBS, the cells were incubated with FITC-conjugated goat anti-pig IgG H&L (1:500 dilution; Abcam, Cambridge, UK) at 37°C for 1 h in dark environment. Finally, the cells were examined using an inverted fluorescence microscope (Nikon, Japan).

### 2.10. Ethic Statements

All animal experiments were approved by the Animal Ethics Committee of Huazhong Agricultural University. The approval number for mouse experiment is HZAUMO-2025-0045. The approval number for pig experiment is HZAUSW-2025-0011. During the experiment, the animals were handled following the ARRIVE guidelines 2.0 [25].

### 2.11. Statistical Analysis

Statistical analyses were performed using GraphPad Prism (version 8.0.1), employing multiple *t*-tests to compare groups. Data are presented as mean ± standard deviation (SD). Statistical significance was determined at *p* < 0.05 (*⁣*^*∗*^), and *p* < 0.01 (*⁣*^*∗∗*^).

## 3. Results

### 3.1. Sample Detection and Isolation of *Lawsonia intracellularis* LI-HuB23

**Figure 1:**
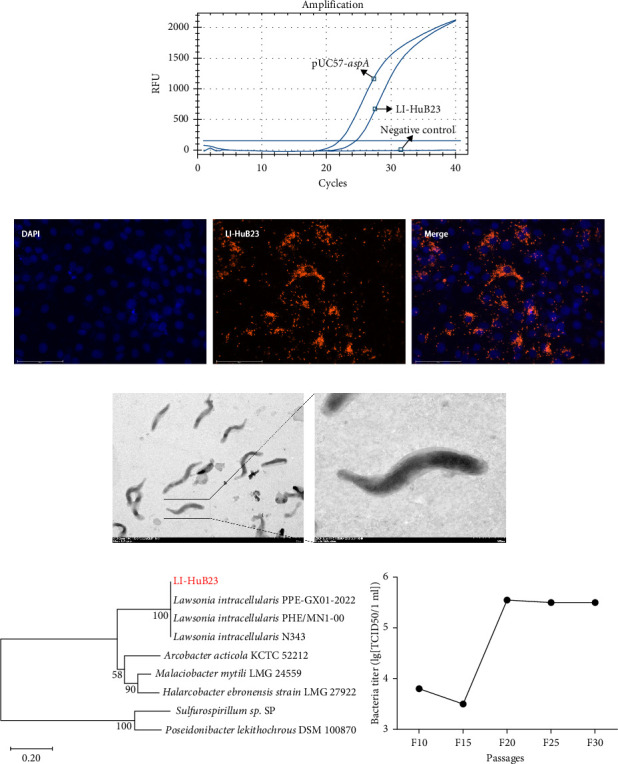
Isolation and characterization of *Lawsonia intracellularis* strain LI-HuB23. (A) qPCR detection of the LI-HuB23 at the 10th passage. (B) IFA of LI-HuB23 at the 10th passage (40x). (C) TEM image showing the morphology of LI-HuB23 (bar = 2 μm). (D) Phylogenetic tree based on nucleotide sequence of *aspA*. (E) Bacterial titers of LI-HuB23 at different passages cultured on IEC-18 cells.

Examination of the 1574 ileal samples collected from the slaughterhouses in Hubei using qPCR, 104 samples were detected to be positive for *L. intracellularis*. By inoculating the homogenates of these 104 samples into IEC-18, a strong positive signals (Ct value = 25) reflecting bacterial multiplication was detected by qPCR in the cells inoculated with one sample (Supporting Information 1: Figure S1) at the 10-passages (Figure 1A). IFA examination demonstrated heavily infected cells (HICs), with more than 30 intracellular *L. intracellularis* organisms per cell (Figure 1B). These results indicated a *L. intracellularis* strain was isolated successfully and it was designated LI-HuB23. TEM examination showed that LI-HuB23 exhibited a rod-shaped morphology (Figure 1C). Phylogenetic analysis based on the nucleotide sequence of aspA showed that LI-HuB23 was phylogenetically related to *L. intracellularis* strains PPE-GX01-2022 (GenBank accession no. CP107054), N343 (GenBank accession no. CP004029), and PHE/MN1-00 (GenBank accession no. NC_008011; Figure 1D and Supporting Information 2: Figure S2). Our on-going experiment revealed that LI-HuB23 could be continuously passaged in IEC-18 over 30-generations (Supporting Information 3: Figure S3). The highest titer of LI-HuB23 was 10^5.55^ TCID_50_/mL in IEC-18 cells (Figure 1E).

### 3.2. Preparation of Monoclonal Antibodies Against *Lawsonia intracellularis* LI-HuB23

**Figure 2:**
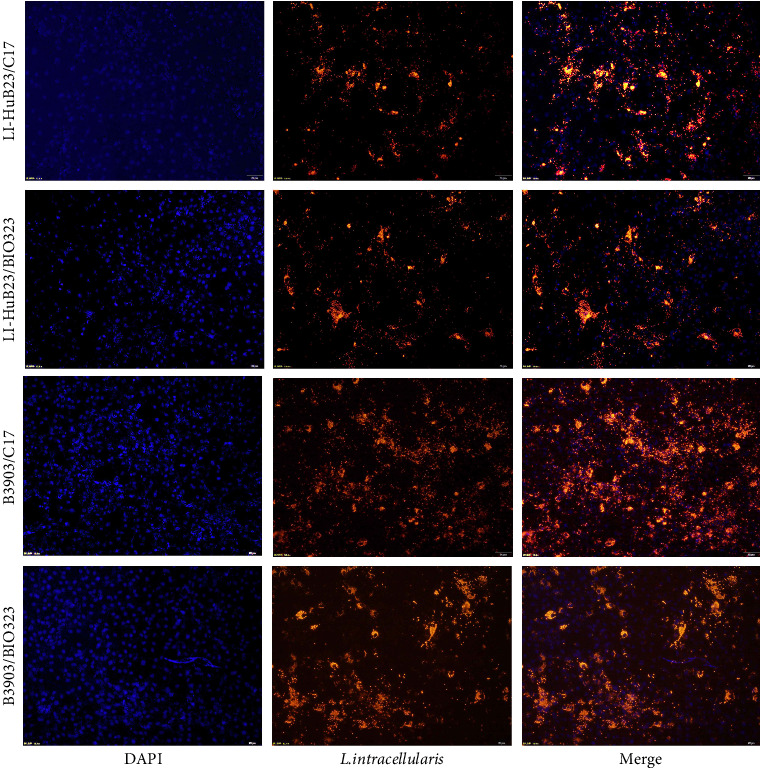
IFA revealing the efficacy of monoclonal antibody C17 on recognizing *Lawsonia intracellularis* strains LI-HuB23 and B3903 compared to that of the commercial monoclonal antibody BIO323.

**Figure 3:**
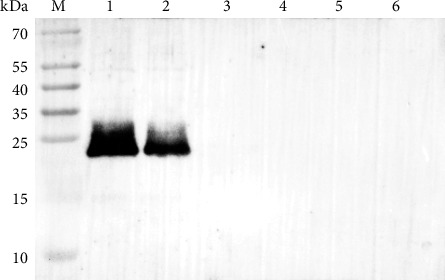
Western blot analysis showing the specificity of monoclonal antibody C17 on recognizing *Lawsoniaintracellularis*. Lane 1: *L. intracellularis* strain B3903; Lane 2: *L. intracellularis* strain LI-HuB23; Lane 3: IEC-18 cells; Lane 4: McCoy cells; Lane 5: *S. choleraesuis*; Lane 6: *E. coli* O157:H7.

A total of three monoclonal antibodies designated A34, C17, and E12 were finally produced by the hybridoma cells and C17 demonstrated the better effect to capture *L. intracellularis* under the same conditions (Supporting Information 4: Figure S4). Particularly, C17 showed a similar effect on recognizing different *L. intracellularis* strains (LI-HuB23 and B3903) with the commercially purchased monoclonal antibody BIO323 (Figure 2). Western blot analysis revealed that C17 exhibited a good specificity to recognize *L. intracellularis* (Figure 3).

### 3.3. Virulence Assessment of *Lawsonia intracellularis* LI-HuB23 in Pigs

**Figure 4:**
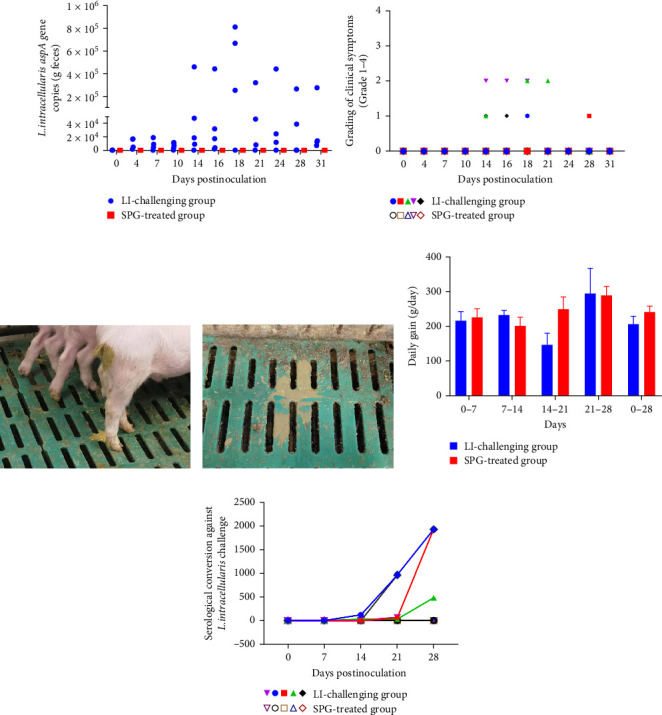
Virulence assessments of *Lawsonia intracellularis* strain LI-HuB23 in pigs. (A) Detection of bacterial DNA in fecal samples from pigs at different days postinoculation using qPCR. (B) Fecal scores of pigs recorded at different days postinoculation. (C) Pig with watery diarrhea. (D) Average daily weight gain in pigs postinoculation. (E) Levels of *L. intracellularis*-specific IgG antibodies in the serum of pigs postinoculation.

After challenging, fecal shedding of *L. intracellularis* was observed on Day 4, with a peak shedding observed Days 14 and 24, reaching a maximum count of 8 × 10^5^ bacterial microorganisms per gram feces (Figure 4A). Additionally, oral inoculation of LI-HuB23 induced the shedding of abnormal feces including soft feces and watery diarrhea between 14 and 28 days after challenging (Figure 4B,C). Pigs inoculated with LI-HuB23 showed an average daily gain (ADG) lowered than the control pigs (206.05 ± 23.48 g/day vs. 241.43 ± 16.78 g/day; Figure 4D). During the experiment, all control pigs were serologically negative for *L. intracellularis* IgG antibodies, while two pigs in the challenging group were serologically positive for *L. intracellularis* IgG at 14 days postinoculation and all pigs were serologically positive at 21 days postinoculation (Figure 4E and Supporting Information 5: Figure S5).

**Figure 5:**
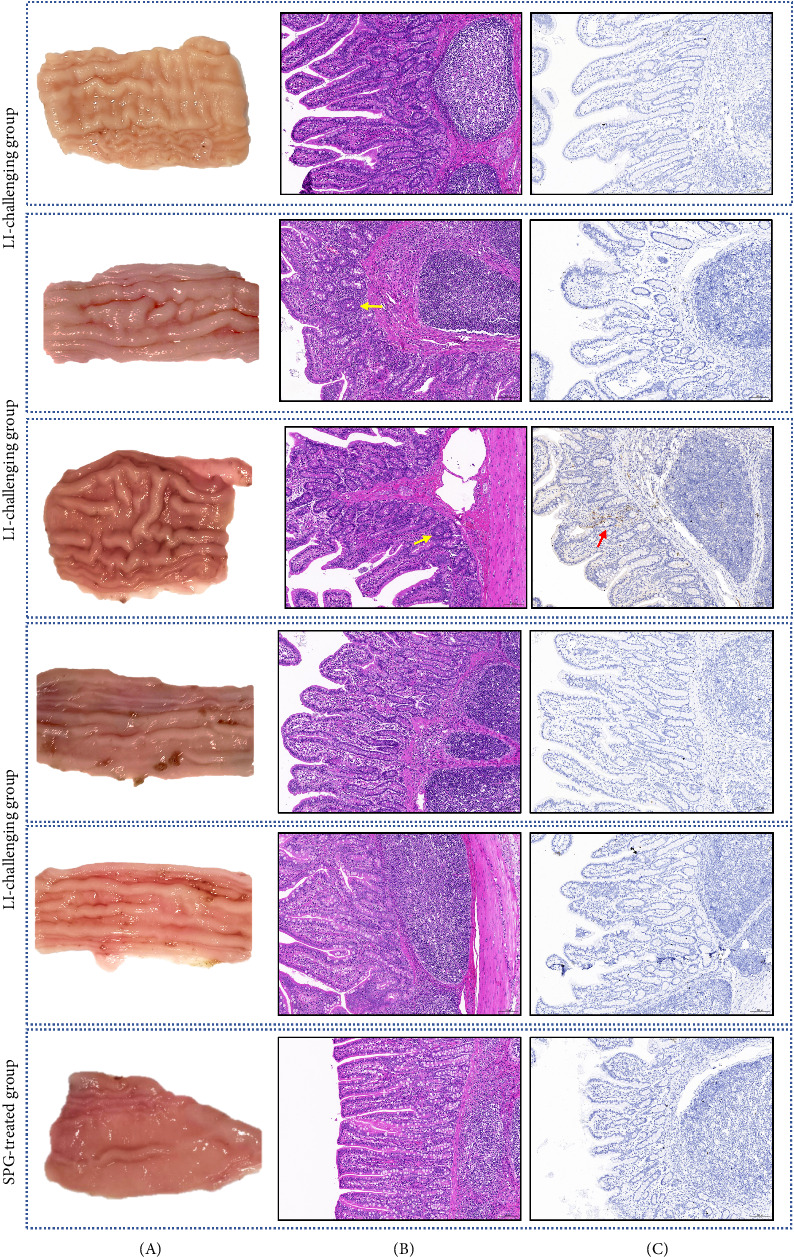
Damages of pig ilea induced by inoculation of *Lawsonia intracellularis* strain LI-HuB23. (A) Intestinal mucosal proliferation observed in the ilea of pigs inoculated with LI-HuB23. (B) Histological examination revealed crypt hyperplasia, characterized reduction in goblet cells within the hyperplastic crypts. Yellow arrow points to the hyperplastic crypts. (C) Colonization of *L. intracellularis* in ileal crypts confirmed by immunohistochemical examination. Red arrow points to the *L. intracellularis* colonizing the ileal crypts.

Intestinal mucosa proliferation was observed in the ilea collected from pigs inoculated with LI-HuB23 (Figure 5A). Histological examination detected crypt hyperplasia, characterized by a reduction in goblet cells within the hyperplastic crypts (Figure 5B). Colonization of *L. intracellularis* in ileal crypts was confirmed by immunohistochemical examination (Figure 5C).

## 4. Discussion

The isolation of *L. intracellularis* remains inherently challenging due to its obligate intracellular and microaerophilic nature [3]. Successful cultivation requires specific host cell systems and highly controlled incubation conditions to maintain the precise atmospheric parameters essential for bacterial growth [18, 26]. Additionally, the complex and heavily contaminated intestinal environment, in which *L. intracellularis* resides further complicates its isolation and propagation [9]. To date, fewer than 25 strains of *L. intracellularis* have been successfully cultured and preserved worldwide [27]. Most of these strains have been derived from cases of porcine hemorrhagic enteropathy (PHE), likely because the number of competing microorganisms in the ileum of infected pigs is relatively low, facilitating the isolation of the bacterium [4]. In China, only a single strain of *L. intracellularis* has been reported from a PHE case and research on the pathogenicity of these isolates in animal models remains limited [13]. In this study, the *L. intracellularis* strain LI-HuB23 was successfully isolated from a porcine intestinal sample obtained at a slaughterhouse. The identity of the isolate was confirmed as *L. intracellularis* at the genetic level through PCR amplification, sequencing, and sequence analysis. TEM and IFA revealed its characteristic curved rod morphology, consistent with previous descriptions [3, 9].

Histological examination of *L. intracellularis* provides a direct visualization of bacterial colonization within cells and tissues, serving as a valuable diagnostic tool for *L. intracellularis* infection. Among commonly used staining methods, Warthin–Starry silver staining has been widely applied [9, 23]. However, its limitations include poor specificity, making it difficult to distinguish *L. intracellularis* from other curved rod-shaped bacteria, as well as inconsistent reproducibility [28]. Antibody-based detection methods, such as IFA, IHC, and IPMA, offer high specificity, superior sensitivity, and strong reproducibility, making them reliable diagnostic tools for *L. intracellularis* infection [28–32]. Previous studies have demonstrated that antibody-based methods exhibit a sensitivity of approximately 87% when compared to the gold standard PCR [10]. In this study, a monoclonal antibody targeting *L. intracellularis* was developed for the specific detection of the bacterium. Western blot analysis confirmed that the antibody specifically recognized a protein of approximately 22 kDa in *L. intracellularis*, with no cross-reactivity observed with IEC-18 cells, McCoy cells, *S. choleraesuis*, or *E. coli* O157:H7. Furthermore, IFA results demonstrated its effectiveness in detecting *L. intracellularis* strains LI-HuB23 and B3903, indicating its potential as a highly specific diagnostic antibody.

During the continuous passage of *L. intracellularis* LI-HuB23, the bacterial titer exhibited an increasing trend and stabilized after the 20th passage, indicating strong adaptation and stable growth in IEC-18 cells. IFA staining revealed HICs, defined by the presence of more than 30 intracellular bacteria per cell. This observation aligns with previous studies describing the intracellular proliferation of *L. intracellularis* within host cells [3, 13, 17, 18]. The TCID_50_ method, a widely utilized approach for quantifying viral titers, is valued for its simplicity, reproducibility, and effectiveness in assessing pathogen infectivity [33]. Although this method does not directly enumerate bacterial cells, it provides an indirect measure of infectivity that correlates with bacterial load, as confirmed by complementary qPCR analysis. Alternative techniques, such as antibody-based staining for bacterial enumeration, present certain limitations, including incomplete bacterial fixation on slides [2, 34], which may lead to underestimation of bacterial counts and reduced reproducibility. Additionally, these methods are unable to differentiate between viable and nonviable bacteria, posing challenges for accurate interpretation of results.

In the animal experiment, 28-day-old pigs were orally inoculated with the 12th passage of LI-HuB23 at a dose of 6.9 × 10^8^*L. intracellularis* (10^6.5^ TCID_50_). Fecal shedding was first observed on Day 4 postinfection, with peak shedding occurring between Days 14 and 24, reaching concentrations of up to 8 × 10^5^*L. intracellularis* per gram of feces. IgG antibodies were detected in some pigs by Day 14 and all pigs were seropositive by Day 21. Vannucci et al. [35] observed similar patterns of fecal shedding and seroconversion in pigs infected with cell-cultured *L. intracellularis*, with peak shedding occurring between Days 14 and 28 and detectable titers by Day 14. Histological analysis of the ileum revealed mild intestinal hyperplasia, with H&E staining showing crypt hyperplasia, epithelial cell layering, and the disappearance of goblet cells in hyperplastic crypts. Immunohistochemistry confirmed the presence of *L. intracellularis* in the crypts, consistent with the characteristic lesions of ileitis [2].

In this study, the intestinal pathological changes observed in pigs infected with the cell culture-derived LI-HuB23 strain were less severe compared to those caused by the clinically derived LI-HuB23 strain. Specifically, gross examination revealed reduced mucosal thickening in the ileum, along with decreased crypt hyperplasia on H&E staining and lower levels of *L. intracellularis* colonization as detected by IHC (Supporting Information S1: Figure S1). This attenuation may be attributed to the strain's adaptation to cell culture through multiple passages. Previous studies, such as those by Vannucci et al. [35] have reported a reduction in virulence following 20–40 passages of strains isolated from PHE cases. The strain used in this study was derived from a porcine intestinal adenomatosis (PIA) case, which typically presents with milder symptoms than PHE, and may, therefore, have undergone more rapid adaptation to cell culture conditions, resulting in reduced pathogenicity.

## 5. Conclusion

In this study, a continuously passaged *L. intracellularis* strain, LI-HuB23, was successfully isolated from 104 *L. intracellularis*-positive ileal samples out of a total of 1574 collected samples. Additionally, a monoclonal antibody with high specificity for *L. intracellularis* was generated. Through continuous passaging, LI-HuB23 exhibited cellular adaptation, maintaining a stable titer over successive passages. Furthermore, infection of 4-week-old pigs with a dose of 6.9 × 10^8^ (10^6.5^ TCID_50_) resulted in clinical symptoms and histopathological lesions characteristic of porcine ileitis. The inoculated pigs exhibited clinical symptoms and histopathological changes consistent with porcine ileitis, thereby confirming the pathogenic potential of LI-HuB23 and its ability to reproduce key features of the disease. These findings provide valuable insights into the in vitro adaptation and pathogenicity of *L. intracellularis*, facilitating future studies on its pathogenesis, immune responses, and vaccine development.

## Data Availability

All the data are provided within the article and the supporting information.

## Supporting Information

Supporting Information 1 Figure S1: Gross appearance, histological examination, and immunohistochemical analysis of the intestinal sample that *L. intracellularis* strain LI-HuB23 was isolated. Yellow arrow points to the hyperplastic crypts, and red arrow points to the *L. intracellularis* colonizing the ileal crypts.

Supporting Information 2 Figure S2: Amino acid sequence alignments of AspA from *L. intracellularis* strains LI-HuB23, PPE-GX01-2022, PHE/MN1-00, and N343.

Supporting Information 3 Figure S3: IFA examination of LI-HuB23 continuously passaged in IEC-18 cells for over 30 generations.

Supporting Information 4 Figure S4: IFA examination of *L. intracellularis* using three monoclonal antibodies (A34, C17, and E12) prepared in this study.

Supporting Information 5 Figure S5: Serological conversion against *L.intracellularis* challenge. (A–E) *L. intracellularis* LI-HuB23-challenging group and (F) SPG-treated group.

## References

[1] McOrist S., Gebhart C. J., Boid R., Barns S. M. (1995). Characterization of *Lawsonia Intracellularis* Gen. Nov., Sp. Nov., the Obligately Intracellular Bacterium of Porcine Proliferative Enteropathy. *International Journal of Systematic Bacteriology*.

[2] McOrist S., Jasni S., Mackie R. A., MacIntyre N., Neef N., Lawson G. H. (1993). Reproduction of Porcine Proliferative Enteropathy With Pure Cultures of Ileal Symbiont Intracellularis. *Infection and Immunity*.

[3] Lawson G. H., McOrist S., Jasni S., Mackie R. A. (1993). Intracellular Bacteria of Porcine Proliferative Enteropathy: Cultivation and Maintenance in Vitro. *Journal of Clinical Microbiology*.

[4] Vannucci F. A., Gebhart C. J., McOrist S. (2019). Proliferative Enteropathy. *Diseases of Swine*.

[5] Lawson G. H., Gebhart C. J. (2000). Proliferative eEnteropathy. *Journal of Comparative Pathology*.

[6] Kroll J. J., Roof M. B., Hoffman L. J., Dickson J. S., Harris D. L. (2005). Proliferative Enteropathy: A Global Enteric Disease of Pigs Caused by *Lawsonia Intracellularis*. *Animal Health Research Reviews*.

[7] Rowan T. G., Lawrence T. L. (1982). Amino Acid Digestibility in Pigs With Signs of Porcine Intestinal Adenomatosis. *Veterinary Record*.

[8] McOrist S. (2005). Defining the Full Costs of Endemic Porcine Proliferative Enteropathy. *The Veterinary Journal*.

[9] Stills H. F. (1991). Isolation of an Intracellular Bacterium From Hamsters (*Mesocricetus Auratus*) With Proliferative Ileitis and Reproduction of the Disease With a Pure Culture. *Infection and Immunity*.

[10] Guedes R. M., Gebhart C. J., Winkelman N. L., Mackie-Nuss R. A., Marsteller T. A., Deen J. (2002). Comparison of Different Methods for Diagnosis of Porcine Proliferative Enteropathy. *Canadian Journal of Veterinary Research*.

[11] Wattanaphansak S., Singer R. S., Gebhart C. J. (2009). In Vitro Antimicrobial Activity Against 10 North American and European *Lawsonia Intracellularis* Isolates. *Veterinary Microbiology*.

[12] Wang L., Wu W., Zhao L. (2024). Fecal PCR Survey and Genome Analysis of *Lawsonia Intracellularis* in China. *Frontiers in Veterinary Science*.

[13] Xiao N., Li J., Li M., Zhou H., Lin H., Fan H. (2022). Isolation and In Vitro Cultivation of *Lawsonia Intracellularis* From China. *Veterinary Microbiology*.

[14] Jones G. F., Ward G. E., Murtaugh M. P., Lin G., Gebhart C. J. (1993). Enhanced Detection of Intracellular Organism of Swine Proliferative Enteritis, Ileal Symbiont Intracellularis, in Feces by Polymerase Chain Reaction. *Journal of Clinical Microbiology*.

[15] Suh D. K., Lym S. K., Bae Y. C., Lee K. W., Choi W. P., Song J. C. (2000). Detection of *Lawsonia Intracellularis* in Diagnostic Specimens by One-Step PCR. *Journal of Veterinary Science*.

[16] Wu W., Wang L., Xie R. (2025). Development and Application of a Triplex Real-Time PCR Method for the Detection of Lawsonia Intracellularis, Brachyspira Hyodysenteriae, and Clostridium Perfringens. *Microbiology Spectrum*.

[17] Yeh J. Y., Kim T. J., Park S. Y. (2006). Isolation of *Lawsonia Intracellularis* in Korea and Reproduction of Proliferative Enteropathy in Pigs and Hamsters. *Journal of Veterinary Medical Science*.

[18] Collins A. M., Swift I., Monckton R. P. (1996). Replication of Australian Porcine Isolates of Ileal Symbiont Intracellularis in Tissue Culture. *Veterinary Microbiology*.

[19] Chen N., Ye M., Huang Y. (2019). Identification of Two Porcine Reproductive and Respiratory Syndrome Virus Variants Sharing High Genomic Homology but With Distinct Virulence. *Viruses*.

[20] Guedes R. M. C., Gebhart C. J. (2003). Preparation and Characterization of Polyclonal and Monoclonal Antibodies Against *Lawsonia Intracellularis*. *Journal of Veterinary Diagnostic Investigation*.

[21] Boesen H. T., Jensen T. K., Jungersen G., Riber U., Boye M., Møller K. (2005). Development, Characterization and Diagnostic Application of a Monoclonal Antibody Specific for a Proteinase K Resistant *Lawsonia Intracellularis* Antigen. *Veterinary Microbiology*.

[22] Reed L. J., Muench H. (1938). A Simple Method of Estimating Fifty Per Cent Endpoints. *American Journal of Epidemiology*.

[23] Joens L. A., Nibbelink S., Glock R. D. (1997). Induction of Gross and Microscopic Lesions of Porcine Proliferative Enteritis by *Lawsonia Intracellularis*. *American Journal of Veterinary Research*.

[24] Guedes R. M. C., Gebhart C. J., Deen J., Winkelman N. L. (2002). Validation of an Immunoperoxidase Monolayer Assay as a Serologic Test for Porcine Proliferative Enteropathy. *Journal of Veterinary Diagnostic Investigation*.

[25] du Sert N. Percie, Ahluwalia A., Alam S. (2020). Reporting Animal Research: Explanation and Elaboration for the ARRIVE guidelines 2.0. *PLOS Biology*.

[26] Vannucci F. A., Wattanaphansak S., Gebhart C. J. (2012). An Alternative Method for Cultivation of *Lawsonia Intracellularis*. *Journal of Clinical Microbiology*.

[27] Wattanaphansak S., Pereira C. E. R., Kaenson W. (2019). Isolation and In Vitro Antimicrobial Susceptibility of Porcine *Lawsonia Intracellularis* From Brazil and Thailand. *BMC Microbiology*.

[28] Huerta B., Arenas A., Carrasco L. (2003). Comparison of Diagnostic Techniques for Porcine Proliferative Enteropathy (*Lawsonia Intracellularis* Infection). *Journal of Comparative Pathology*.

[29] Kim J., Choi C., Cho W.-S., Chae C. (2000). Immunohistochemistry and Polymerase Chain Reaction for the Detection of *Lawsonia Intracellularis* in Porcine Intestinal Tissues With Proliferative Enteropathy. *Journal of Veterinary Medical Science*.

[30] Szczotka A., Stadejek T., Żmudzki J., Nowak A., Osiński Z., Pejsak Z. (2011). Immunohistochemical Detection of *Lawsonia Intracellularis* in Tissue Sections From Pigs. *Polish Journal of Veterinary Sciences*.

[31] Jensen T. K., Boesen H. T., Vigre H., Boye M. (2010). Detection of *Lawsonia Intracellularis* in Formalin-Fixed Porcine Intestinal Tissue Samples: Comparison of Immunofluorescence and In-Situ Hybridization, and Evaluation of the Effects of Controlled Autolysis. *Journal of Comparative Pathology*.

[32] Ladinig A., Sommerfeld-Stur I., Weissenböck H. (2009). Comparative Evaluation of Diagnostic Methods for Lawsonia intracellularis Infection in Pigs, With Emphasis on Cases Lacking Characteristic Lesions. *Journal of Comparative Pathology*.

[33] Baid K., Banerjee A., Chiok K. R., Thakur A. (2024). Median Tissue Culture Infectious Dose 50 (TCID(50)) Assay to Determine Infectivity of Cytopathic Viruses. *Methods in Molecular Biology*.

[34] Guedes R. M. C., Gebhart C. J. (2003). Onset and Duration of Fecal Shedding, Cell-Mediated and Humoral Immune Responses in Pigs After Challenge With a Pathogenic Isolate or Attenuated Vaccine Strain of *Lawsonia Intracellularis*. *Veterinary Microbiology*.

[35] Vannucci F. A., Beckler D., Pusterla N., Mapes S. M., Gebhart C. J. (2013). Attenuation of Virulence of *Lawsonia Intracellularis* After In Vitro Passages and Its Effects on the Experimental Reproduction of Porcine Proliferative Enteropathy. *Veterinary Microbiology*.

