# Supplementary figures and images for: Isolation, Passage, and Pathogenicity of a Newly Isolated Lawsonia intracellularis Strain From Hubei, China
Source: Transbound Emerg Dis. 2025 Jul 1;2025:2501719. doi: 10.1155/tbed/2501719 (PMC12237556; doi:10.1155/tbed/2501719)

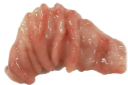

**Gross Appearance**

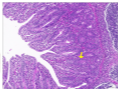

**H&E**

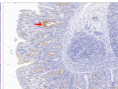

**IHC**

Supplement: Supporting Information 1 — Figure S1: Gross appearance, histological examination, and immunohistochemical analysis of the intestinal sample that L. intracellularis strain LI-HuB23 was isolated. Yellow arrow points to the hyperplastic crypts, and red arrow points to the L. intracellularis colonizing the ileal crypts. [file 2501719.f1.pdf]

## Slide 1
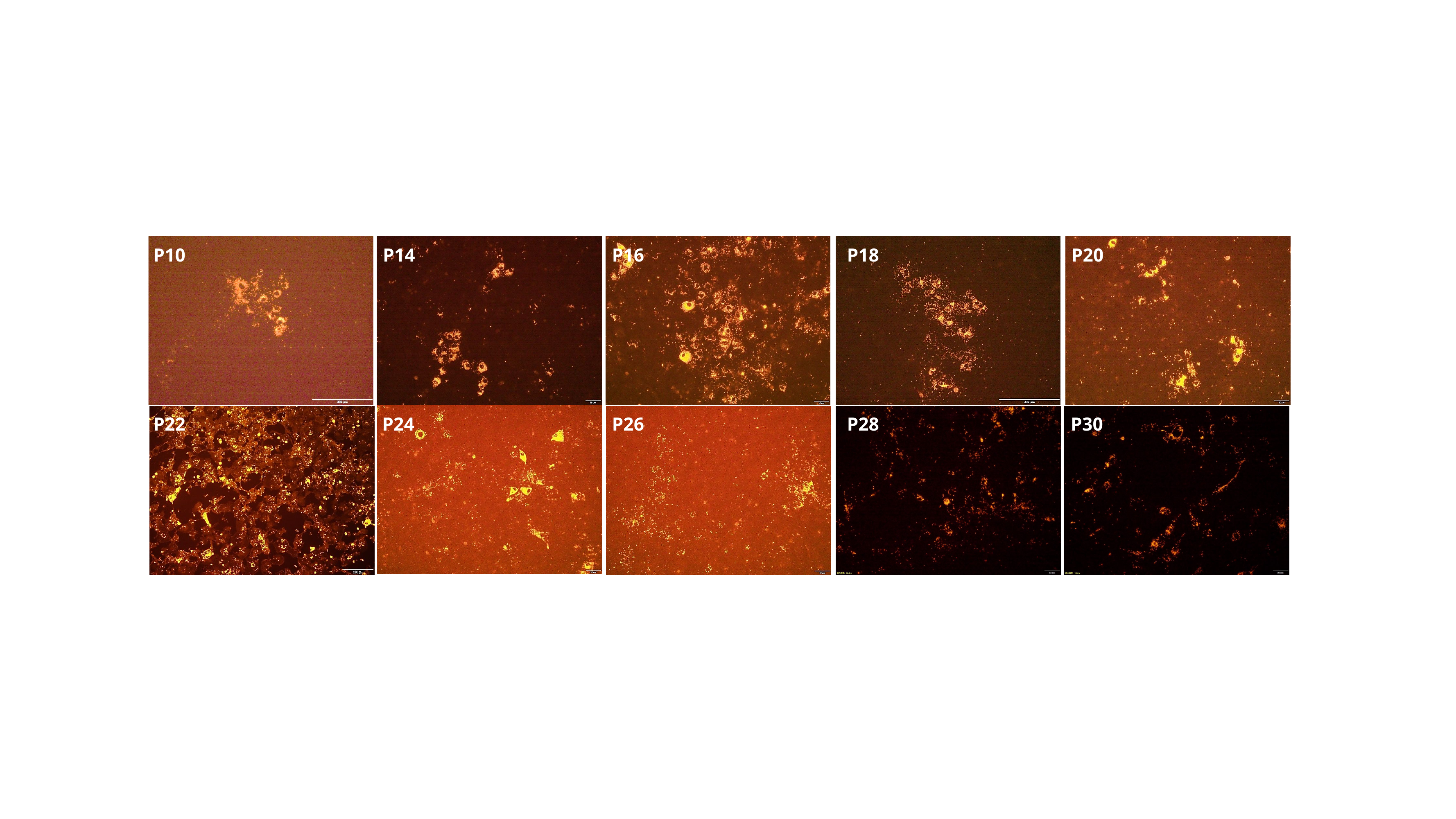

P14
 P18
P20
P10
 P16
P24
P22
 P26
 P28
 P30

Supplement: Supporting Information 3 — Figure S3: IFA examination of LI-HuB23 continuously passaged in IEC-18 cells for over 30 generations. [file 2501719.f3.pptx]

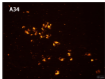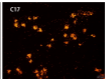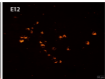

Supplement: Supporting Information 4 — Figure S4: IFA examination of L. intracellularis using three monoclonal antibodies (A34, C17, and E12) prepared in this study. [file 2501719.f4.pdf]

## Slide 1
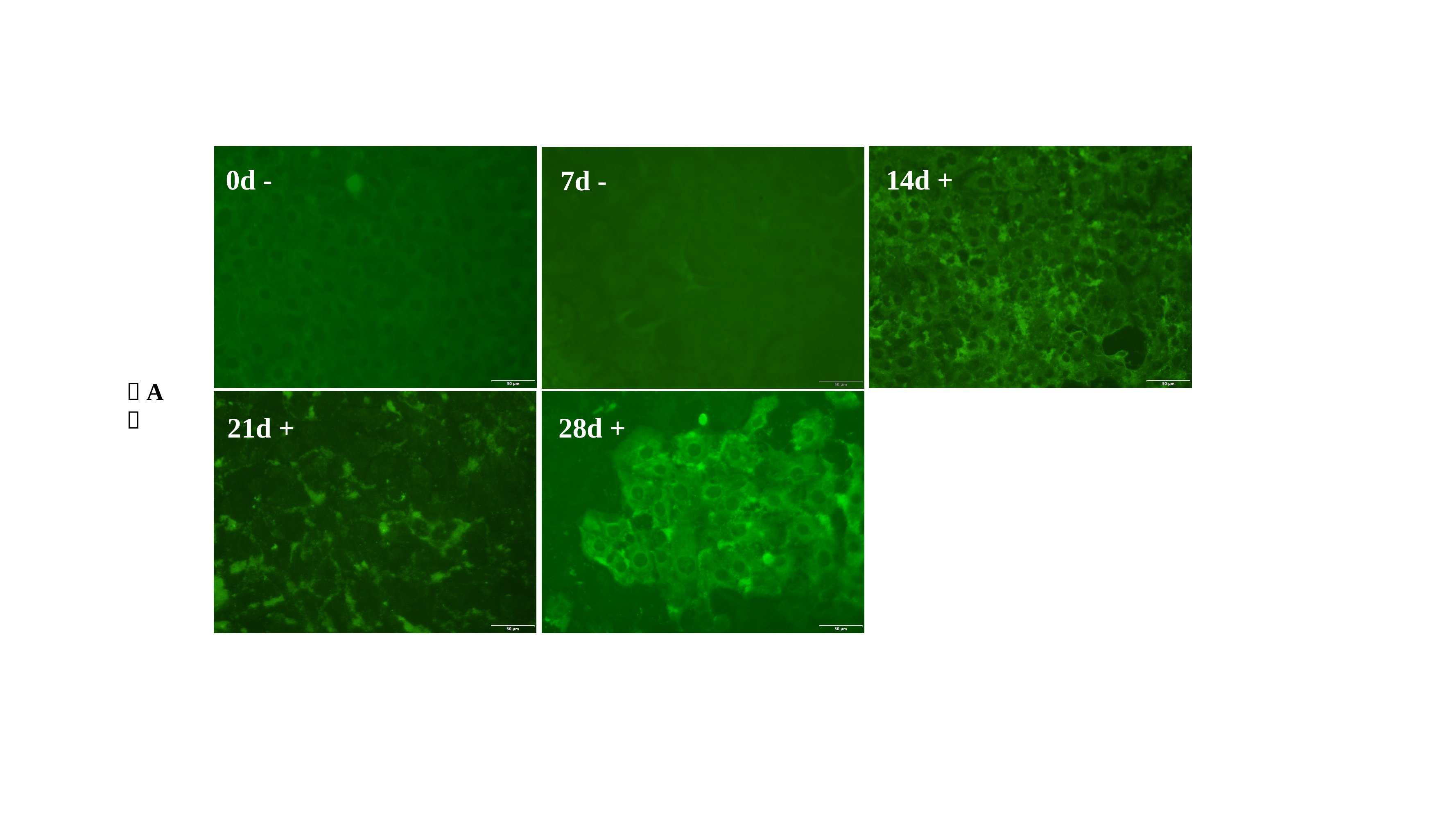

14d +
0d -
7d -
21d +
28d +
（A）

## Slide 2
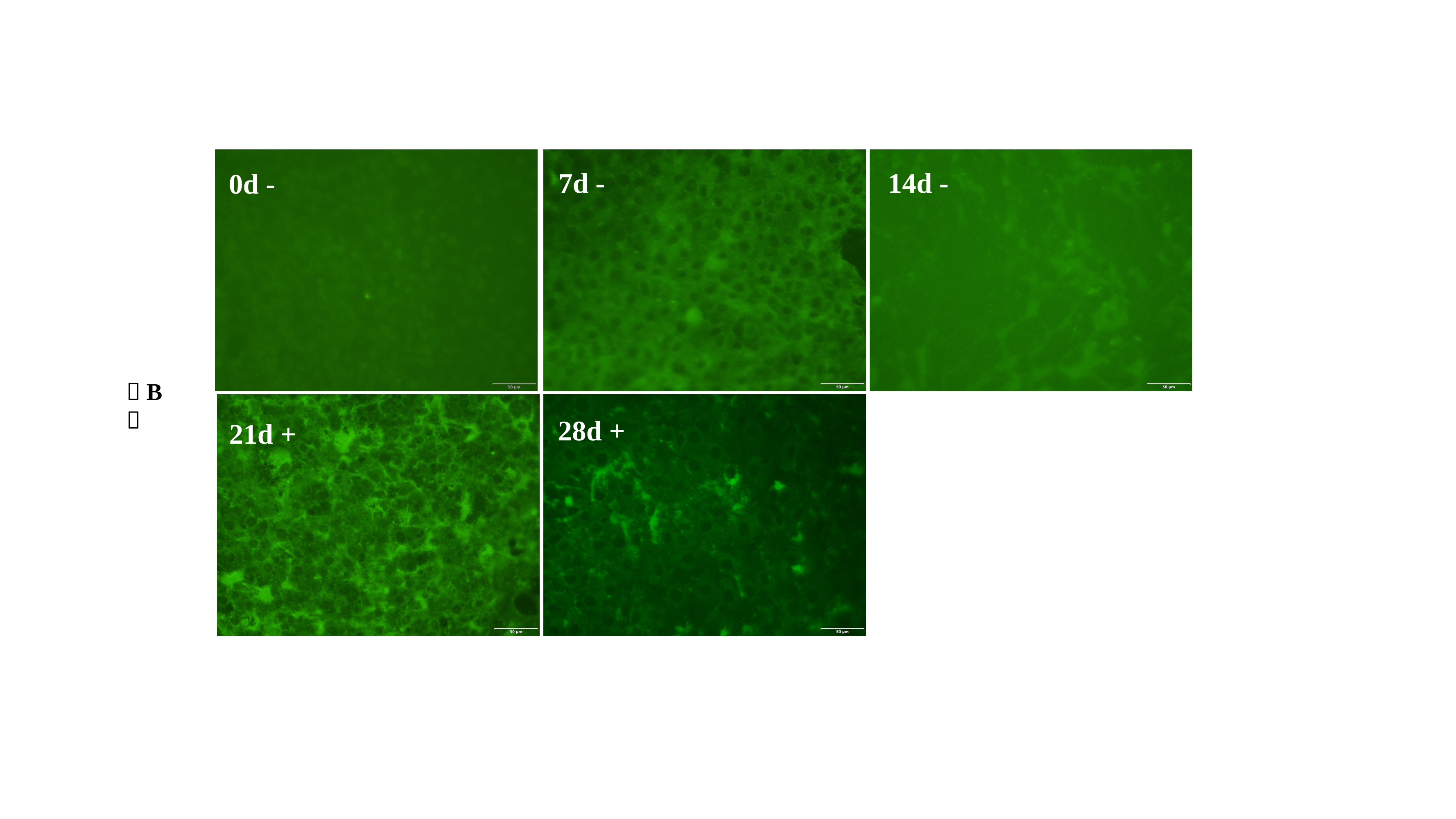

7d -
14d -
0d -
28d +
21d +
（B）

## Slide 3
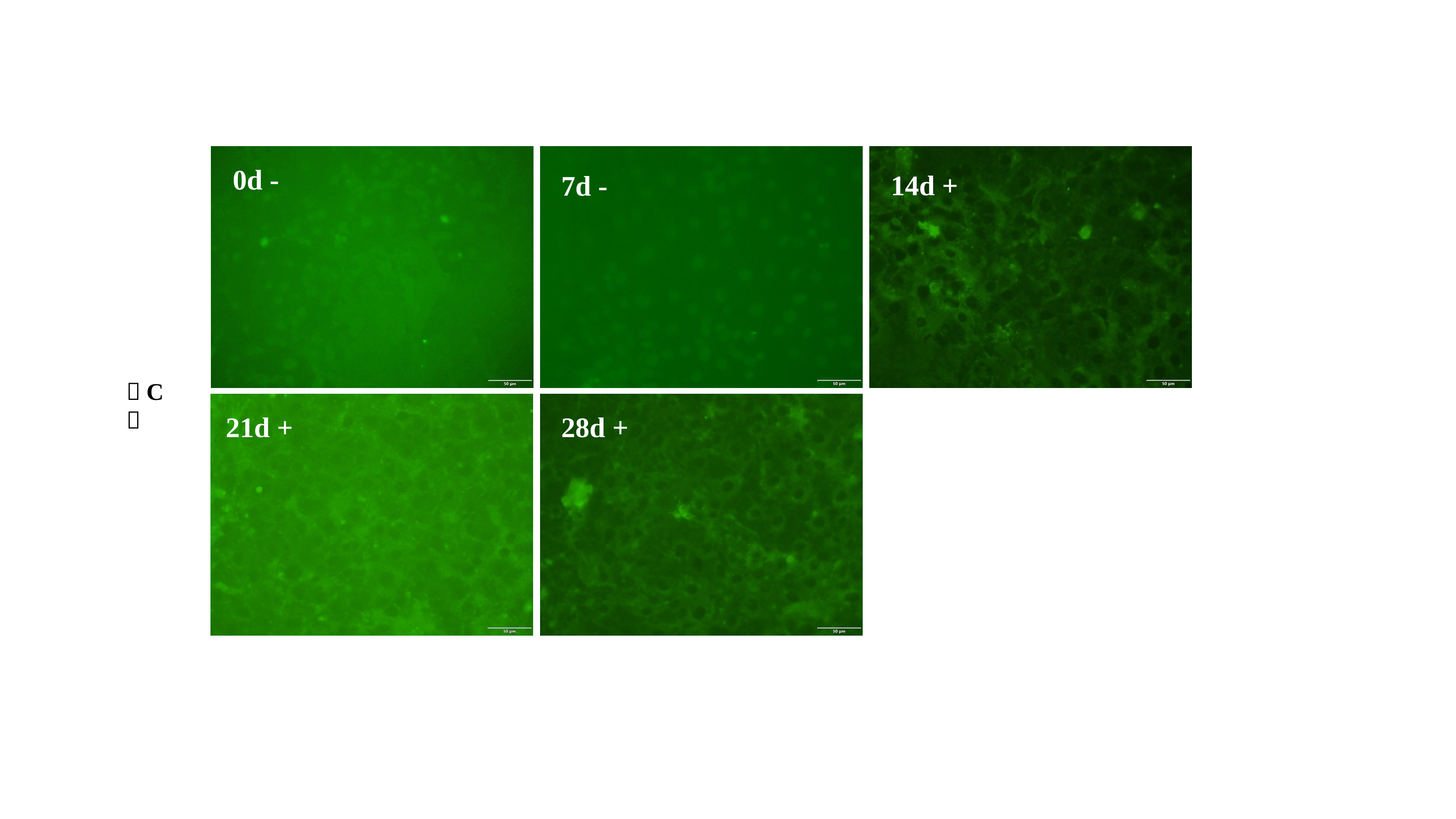

0d -
14d +
7d -
21d +
28d +
（C）

## Slide 4
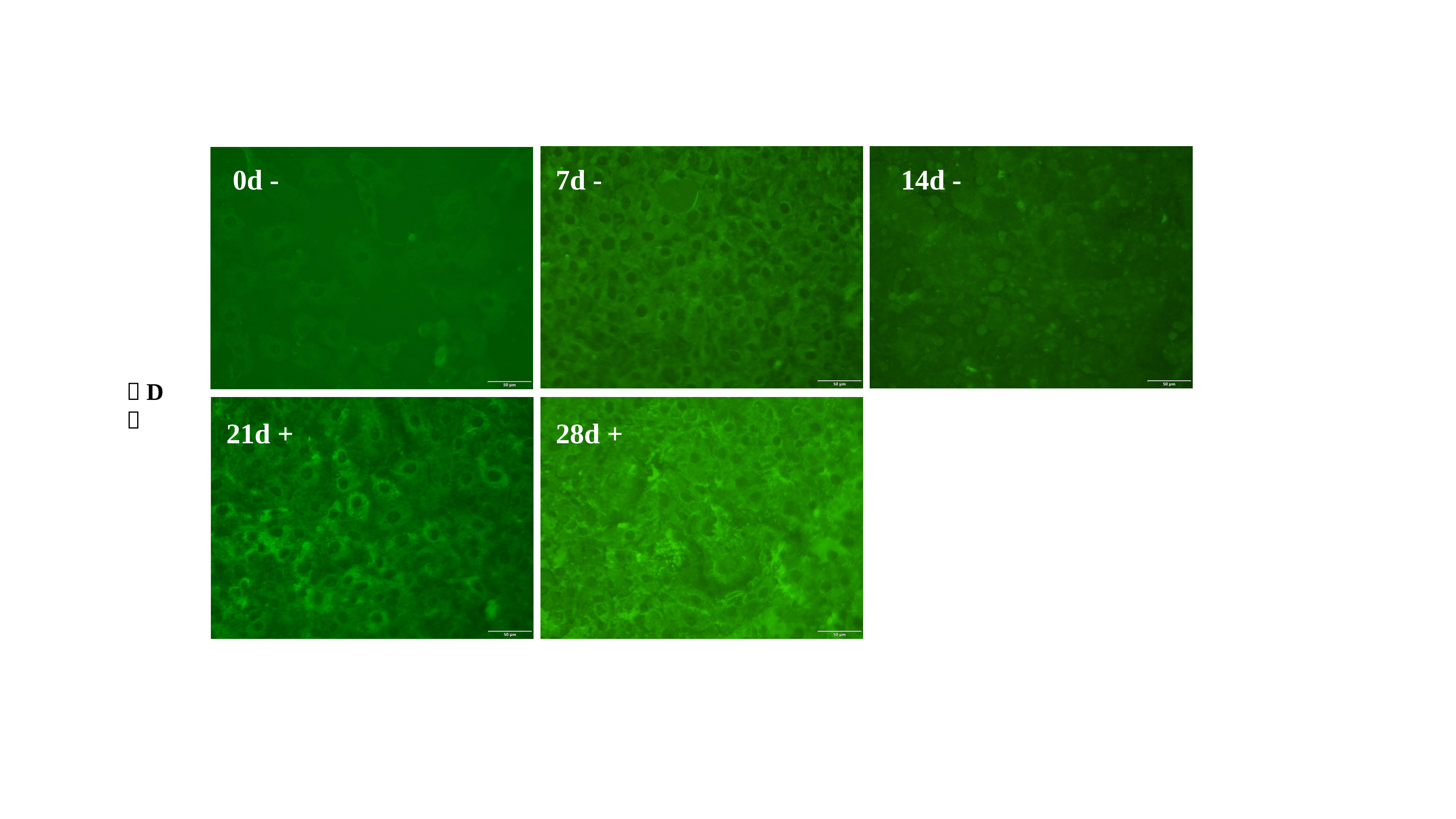

0d -
7d -
14d -
21d +
28d +
（D）

## Slide 5
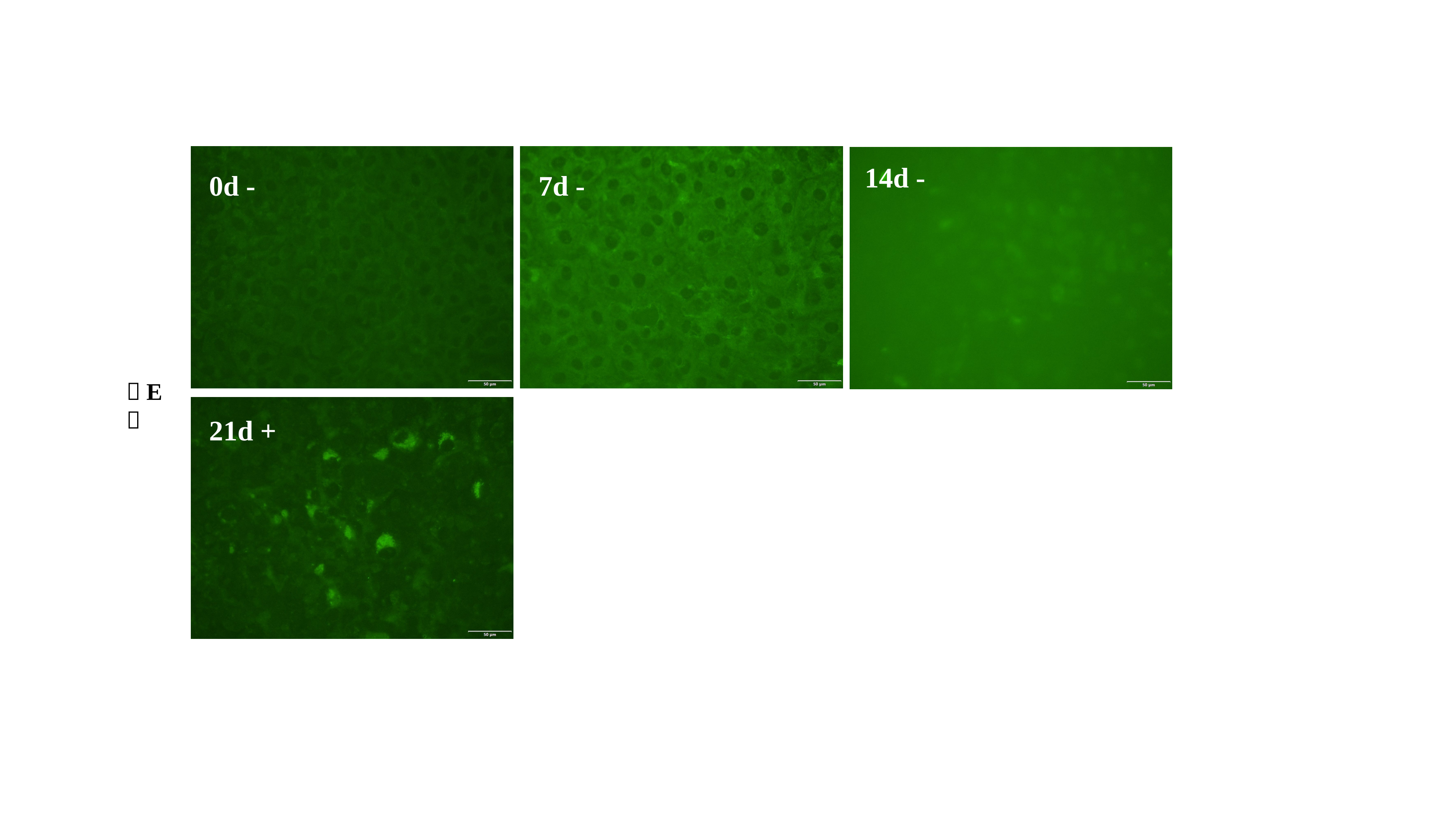

14d -
0d -
7d -
21d +
（E）

## Slide 6
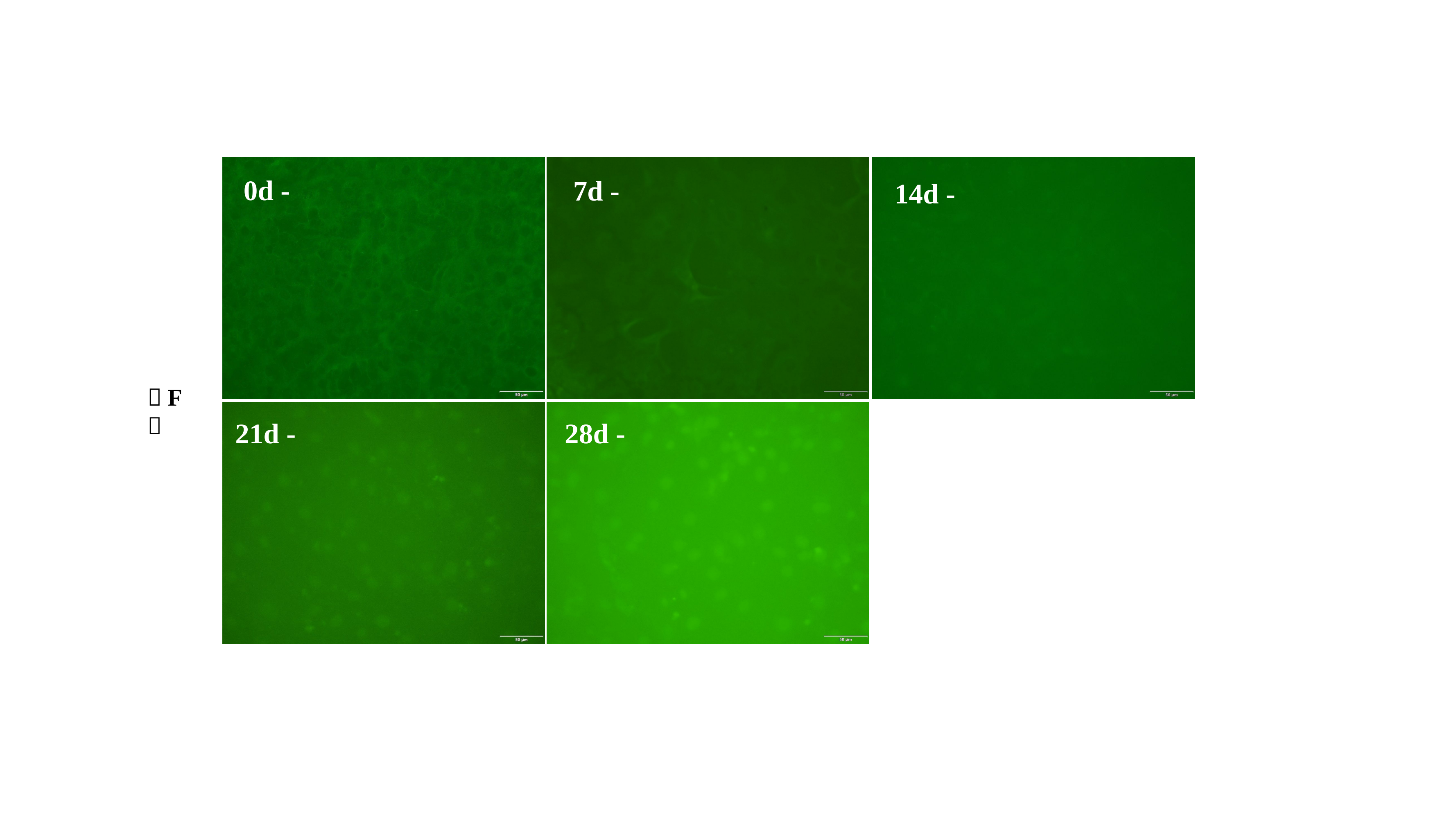

0d -
7d -
14d -
（F）
21d -
28d -

Supplement: Supporting Information 5 — Figure S5: Serological conversion against L.intracellularis challenge. (A–E) L. intracellularis LI-HuB23-challenging group and (F) SPG-treated group. [file 2501719.f5.pptx]
