# Supplementary material for: Isolation, Passage, and Pathogenicity of a Newly Isolated Lawsonia intracellularis Strain From Hubei, China
Source: Transbound Emerg Dis. 2025 Jul 1;2025:2501719. doi: 10.1155/tbed/2501719 (PMC12237556; doi:10.1155/tbed/2501719)
Supplement: Supporting Information 2 — Figure S2: Amino acid sequence alignments of AspA from L. intracellularis strains LI-HuB23, PPE-GX01-2022, PHE/MN1-00, and N343. [file 2501719.f2.pdf]

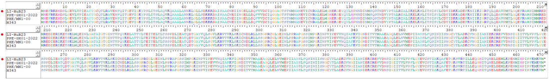
LI-HuB23
PPE-GX01-2022
PHE/MN1-00
N343
